# Synopsis of Barrier Function of Skin and Oral Mucosa—Volume 1

**DOI:** 10.3390/ijms22179383

**Published:** 2021-08-30

**Authors:** Philip W. Wertz

**Affiliations:** University of Iowa, Iowa City, IA 52242, USA; philipwwertz@gmail.com

This is an attempt to make readers of the second edition of *International Journal of Molecular Sciences* Special Issue on the Barrier Function of Skin and Oral Mucosa aware of the content of the first edition on this same topic. Some highlights are noted.

The roles of lipids in the barrier function of skin and advanced methods for permeability enhancement for the purpose of transdermal drug delivery were reviewed [1]. The permeability barrier of the skin is dependent upon the state of organization of ceramides, cholesterol and free fatty acids in the stratum corneum. The keratinized oral regions of the gingiva and palate are similar to skin, while the nonkeratinized floor of the mouth and buccal regions do not produce a stratum corneum. There is some evidence that ceramides, cholesterol and fatty acids may be important for barrier function of these regions. Advanced methods of transdermal drug delivery and buccal absorption are discussed. The roles of lipid in the keratinized and nonkeratinized regions of the oral mucosa, buccal absorption, microbiological barriers and immune barriers were also discussed [1,2]. The physical barrier and the interaction between the oral microbiota and the immune system are considered.

Several studies demonstrated the dependence of skin barrier function on various physical aspects of the stratum corneum (SC) lipids. One study demonstrated that the repeat distance of the long lamellar phase depends upon the chain length of the free fatty acids [3]. Normally, the free fatty acids in healthy stratum corneum are in the range of C20:0 through C28:0, and the repeat distance is about 13 nm. When the fatty acids are C16:0 and C18:0 the periodicity is shorter. The shorter fatty acids are present in SC in some inflammatory conditions and barrier function is impaired. Another study examined the effects of the stereochemistry of the α-hydroxyl group in α-hydroxyacid-containing ceramides on a range of physical properties of model SC membranes [4]. Ceramides with the unnatural S configuration were compared with ceramides possessing the natural R configuration in ceramides AS, AdS and AP. There was no general consequence of the stereochemistry. The results were dependent on a combination of the sphingoid base, the stereochemistry at the α-carbon and the measure of permeability barrier function that was used.

Several manuscripts examined methods to overcome the skin barrier for the purpose of drug delivery. One of these reviewed the literature on lipid-based nanosystems [5]. This included liposomes, ethosomes, transfersomes, niosomes. nanostructured lipid carriers, solid lipid nanoparticles, cubosomes and monoolean aqueous dispersions. One manuscript described the effective delivery of desoximetasone by incorporation of the corticosteroid into niosomes [6]. Such formulations could be useful in treatment of a number of skin diseases including psoriasis.

One review discussed the effects of different wavelengths of light on skin [7]. Deleterious effects of sunlight were discussed, but the major emphasis was the benefits of laser irradiation for treatment of various skin diseases or conditions. The diseases treated with laser irradiation included acne, atopic dermatitis and *Candida albicans* infection. Laser treatment can also promote skin wound healing. The ruby laser is used for removal of birth marks and tattoos.

Air pollution, including fine particulate material, is damaging to the skin barrier. A multimodal nonlinear optical imaging system has been described to visualize the deposition and penetration of particulate matter into human skin [8].

The skin barrier function is impaired in a variety of inflammatory skin diseases including atopic dermatitis and psoriasis. Strategies for developing optimal formulations for topical therapy of such conditions have been presented [9]. The importance of the physical properties of active agents is discussed. Recommended base components include sorbitol and glycerol for moisturization, the human sebum components squalene and triglycerides for a thin occlusive lipid film, silicon oil for occlusion and linoleic acid as a PPARγ agonist.

A major causative factor in skin cancer is UV radiation. Other life-style factors that influence skin carcinogenesis include age, smoking, alcohol consumption, dietary fiber, obesity and circadian rhythm disruption [10]. Circadian rhythm is under genetic control [11]. The susceptibility of DNA in skin cells as well as the expression of DNA repair enzymes vary as a function of time in a cyclic manner [11]. 

Several articles dealt with skin problems relating to aging. 11β-hydroxysteroid dehydrogenase type 1 was shown to be at higher levels in human stratum corneum and oral epithelium compared to younger subjects [12]. The resulting increase in cortisol could cause deterioration of the skin barrier function. Aged mice had greater active glucocorticoid levels than young mice, and also had higher transepidermal water loss. Rosacea is a disease that causes redness and visible blood vesicles in the face. It is most commonly seen in middle aged women. The Hippo pathway has two components, YAP and TAZ, and is involved in angiogenesis [13]. The expression levels of YAP and TAZ were found to be elevated in subjects with rosacea. In a rosacea-like mouse model, the clinical features of rosacea were seen to improve after injection of a YAP/TAZ inhibitor. The pathophysiology and treatment of pruritis in elderly skin has been discussed [14]. There are many factors that may contribute to itch in elderly skin, but one of these may be decreased lamellar granule secretion and reduced intercellular lipids in the stratum corneum. Extramammary Paget’s disease (EMPD) is a cancer that arises in apocrine gland-rich areas of the skin, primarily in elderly people [15]. This study demonstrated that this tumor expresses a high level of trophoblast cell surface antigen 2, as do some other solid tumors. Recently, a drug-conjugated antibody to Trop2 has been tested with positive results against several other solid tumors. This suggests that a similar approach may be useful for treatment of EMPD.

In one study, barrier function of cultured human skin equivalents was optimized by adjusting three factors [16]. It had been shown that incorporating chitosan into the dermal matrix, reducing the oxygen level and inhibiting the lipid X receptor improved barrier function in human skin equivalents. This study optimized these variables. The result was a human skin equivalent with stratum corneum with lipid composition and organization that more closely resembled what is found in normal human stratum corneum. The barrier function was significantly improved.

In another study, human oral mucosal stem cells were isolated and grown in supplemented hormone epithelial medium [17]. The cells expressed markers indicative of differentiation toward corneal determination. Further studies are needed, but this could provide a tool for tissue engineering to produce corneal replacements.

Autophagy plays an important role in epidermal homeostasis [18]. This includes maintaining the proper barrier function of the skin. Regulation of autophagy may provide a means for treatment of skin barrier dysfunction. Autophagy and skin barrier disorders is discussed, and potential therapeutic strategies are suggested.
